# Risk Factors for Fetal Death and Maternal AP Severity in Acute Pancreatitis in Pregnancy

**DOI:** 10.3389/fped.2021.769400

**Published:** 2021-12-02

**Authors:** Xiaolei Shi, Yuepeng Hu, Na Pu, Guofu Zhang, Jingzhu Zhang, Jing Zhou, Bo Ye, Gang Li, Lu Ke, Yuxiu Liu, Qi Yang, Zhihui Tong, Weiqin Li

**Affiliations:** Department of Critical Care Medicine, Jinling Hospital, Medical School of Nanjing University, Nanjing, China

**Keywords:** acute pancreatitis in pregnancy, hypertriglyceridemia, fetal intrauterine death, ARDS, gestational week

## Abstract

**Background:** Acute pancreatitis in pregnancy is a rare but highly life-threatening gestational and perinatal disease.

**Objective:** This study aimed to identify the risk factors for fetal death and acute pancreatitis severity.

**Methods:** This retrospective cohort study enrolled patients with acute pancreatitis in pregnancy in our center from January 1, 2012, to August 1, 2020, and classified them according to two clinical endpoints, fetal outcome and disease severity. The groups were examined and compared according to gestational week, etiology, gravidity and parity, complications in pre- and post-delivery, and medical history. Logistic regression analysis was performed to identify the independent risk factors for fetal death and acute pancreatitis severity.

**Results:** Of the 90 enrolled patients, 28 (31.1%) had fetal death and 43 (47.8%) had severe acute pancreatitis. Logistic regression analysis showed that pre-delivery acute respiratory distress syndrome (OR, 5.8; 95% CI, 1.5–22.4; *p* = 0.010) and gestational week (OR, 0.9; 95% CI, 0.8–1.0; *p* = 0.011) were risk factors for fetal death. Gestation week (OR, 1.2; 95% CI, 1.1–1.3; *p* = 0.003) and fetal intrauterine death (OR, 5.9; 95% CI, 1.8–19.4; *p* = 0.003) were risk factors for severe acute pancreatitis.

**Conclusions:** Pre-delivery acute respiratory distress syndrome and gestational week were independent risk factors for fetal death. Fetal intrauterine death and gestational week were independent risk factors for severe acute pancreatitis.

## Introduction

Acute pancreatitis in pregnancy (APIP) refers to acute pancreatitis that occurs during pregnancy through 3 months after delivery. APIP is a rare but highly life-threatening gestational and perinatal disease. Recently, the worldwide reported incidence was approximately 1/1,000–1/12,000 (1, 2) with mortality around ~0.97 and ~4.65% for mothers and fetuses (1, 3–6), respectively. However, mortality is much higher in China (~5.13% and 9–20%, respectively) (2, 7–9). In comparison, preeclampsia/eclampsia, one of the most common diseases in pregnancy with an incidence of 4.6–19.4%, has a similar mortality rate to APIP, at 0.4–13.5% and 7.3–15% in the mother and fetus, respectively (10–12).

Many studies have reported that hypertriglyceridemia, maternal age, number of pregnancies, high-fat diet, body mass index (BMI), gestational trimester, delayed diagnosis, and gestational complications are risk factors for poor maternal and fetal outcomes (1, 3, 8, 13–17). However, previous reports have analyzed the risk factors of fetal death associated with the entire course of acute pancreatitis (AP) but did not divide fetal death data into pre- and post-delivery although fetal death includes intrauterine death and death after delivery. However, Xu et al. and Jin et al. have reported that intrauterine fetal death is the main type of fetal death associated with APIP where all perinatal losses were due to spontaneous abortion or demise without any cases of fetal death after delivery (7, 13). Therefore, more evidence is needed on the risk factors of fetal intrauterine death analyzed in the pre-delivery period, in addition to the entire course of AP.

Herein, we conducted an 8-year single-center retrospective study and analyzed the risk factors of fetal intrauterine death in the pre-delivery period and maternal AP severity in the entire AP course, which might help in early recognition and guide us to provide advanced intervention.

## Materials and Methods

### Patient Selection and Clinical Data

This 8-year (from January 2012 to August 2020), single-center, retrospective cohort study was conducted at the Center of Severe Acute Pancreatitis, Jinling Hospital. Informed consent involving data storage and publication was obtained during hospitalization from each patient in the database.

The clinical information collected included maternal age, gestational age at AP onset and at delivery, etiology of AP, disease severity, preliminary management, maternal and fetal outcomes, local and systematic complications, medical history, gravidity and parity, and family medical history. Patients who developed AP during the postpartum period were excluded.

### Diagnostic Criteria

We examined cases of AP occurring during the gestational and the perinatal period. The AP diagnosis was based on the Atlanta classification, determined by at least two of the following: (1) acute upper abdominal pain radiating to the back, (2) serum amylase or lipase level three times higher than normal, and (3) radiological evidence indicating AP (18, 19).

By etiology, APIP can be categorized into biliary APIP (B-APIP), hypertriglyceridemia-induced APIP (HTG-APIP), or other causes such as alcohol use. B-APIP was diagnosed by an increase in alanine aminotransferase level > 150 U/L within 48 h, as well as radiological evidence from abdominal ultrasonography and magnetic resonance cholangiopancreatography (19, 20). HTG-AP was diagnosed by a serum triglyceride level of more than 11.3 mmol/L or between 5.65 and 11.3 mmol/L with a lipid turbidity appearance after excluding gallstone, alcohol, or medication factors (21). According to the criteria, APIP patients were divided into three etiologies: B-APIP, HTG-APIP, and other kinds of APIP.

The severity of APIP was divided into three categories, mild AP (MAP), moderate-severe AP (MSAP), and severe AP (SAP), according to the Revised Atlanta criteria (22). MAP is defined as AP without organ dysfunction or generalized complications. MSAP is defined as AP with transient organ dysfunction or localized/generalized complications within 48 h after treatment. SAP is defined as AP with persistent organ dysfunction or localized/generalized complications for more than 48 h after treatment. The localized complications were evaluated by contrast-enhanced computed tomography (CT) or magnetic resonance imaging (MRI), and all were performed after delivery. Fetal intrauterine death was defined as the absence of fetal movement on self-monitoring, and the baseline fetal heartbeat was no beats per minute. All fetal deaths were defined as fetal intrauterine deaths. Trimester categorization was defined as first trimester (1–12 weeks), second trimester (13–28 weeks), and third trimester (≥29 weeks) (20).

### Classification and Analysis of Acute Pancreatitis in Pregnancy

For analysis of APIP, we divided the patients into two groups based on fetal death or survival, with 28 and 62 patients, respectively, and distinguished complications into pre- and post-delivery periods. In the pre-delivery period, maternal complications were indicated by clinical symptoms but without radiological evaluation, and APIP disease severity could not be established. The risk factors of fetal death were analyzed by many clinical features, such as maternal age, the gestational week at AP onset, AP etiology, medical history, gravidity and parity, and pre-delivery complications [including acute respiratory distress syndrome (ARDS), acute kidney injury (AKI), acidosis, and shock].

In addition, the risk factors for the severity of AP included clinical features such as maternal age, the gestational week at AP onset, AP etiology, medical history, gravidity and parity, and fetal intrauterine death.

### Statistical Analysis

Assessment of normality for continuous variables was performed using the Kolmogorov–Smirnov test and visual inspection of histograms. Continuous variables are presented as mean ± SD when normally distributed or as medians and interquartile ranges when non-normally distributed. Categorical variables are described as frequencies and percentages. Student *t*-tests were used to compare the differences between two groups of normally distributed variables and the Mann–Whitney *U*-test for non-normally distributed variables. Pearson chi-square tests or Fisher's exact tests were used to compare categorical variables, as appropriate. Variables with a probability value of *p* < 0.10 in the univariable analysis were entered into the multivariable logistic regression model, which used the backward method to establish whether they were independently associated with the clinical endpoints of fetal outcome and AP severity. Also, a goodness fit of the model was performed by the Hosmer and Lemeshow test. A *p-value* of 0.05 or less was considered statistically significant. All statistical analyses were performed using SPSS software (version 22.0; SPSS Statistics, Chicago, IL, USA).

## Results

### Clinical Features of Acute Pancreatitis in Pregnancy and Study Flowchart

**Figure 1:**
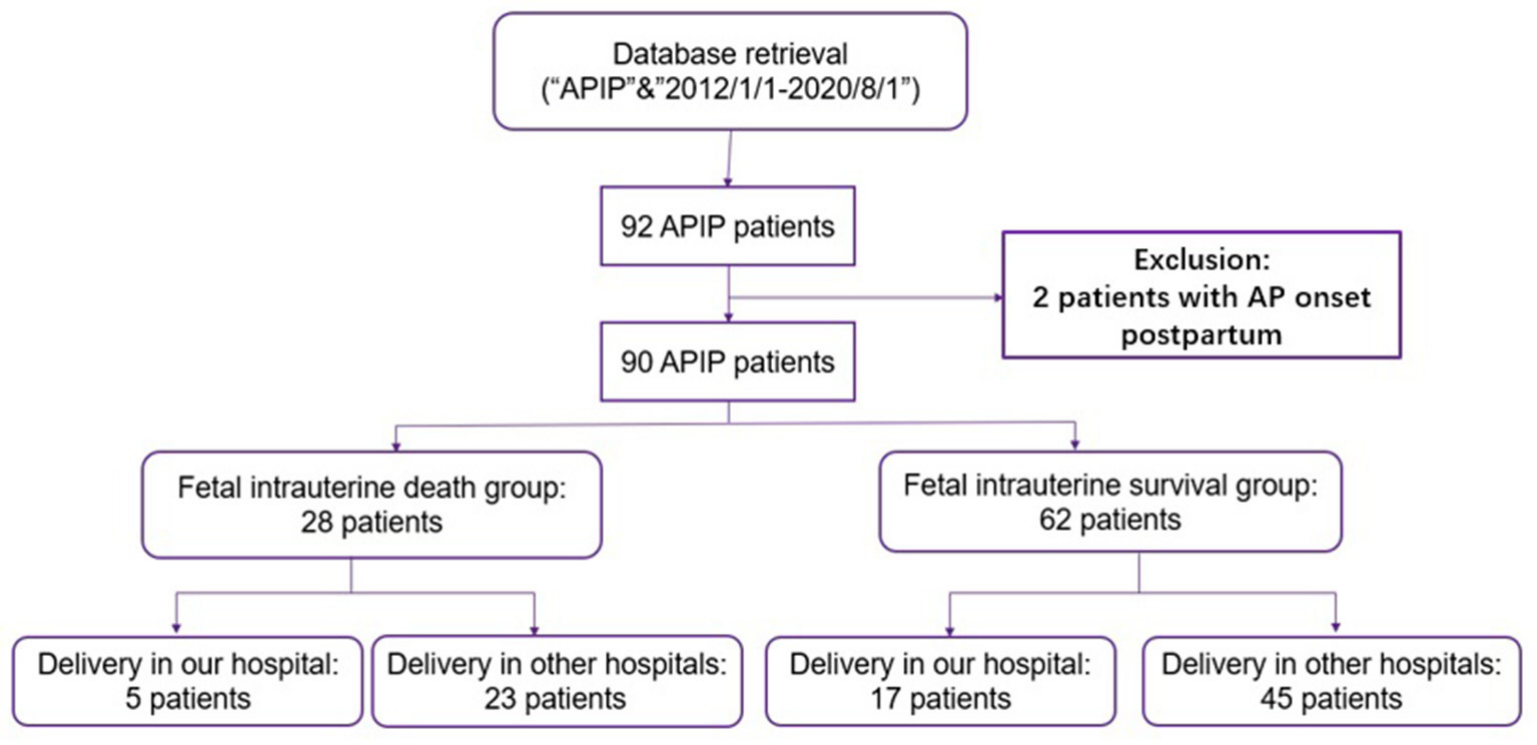
The flowchart of the inclusion and exclusion criteria. APIP, acute pancreatitis in pregnancy.

From January 1, 2012 to August 1, 2020, 92 APIP patients were at our center for treatment. In total, 90 APIP patients were enrolled in this study, excluding two patients with AP onset in the postpartum period. As shown in Figure 1, the patients were divided into two groups: fetal death (28 patients) and fetal survival (62 patients). In the fetal death group, all fetal deaths were fetal intrauterine deaths, including five fetal deaths after transfer to our center and 23 fetal deaths before transfer.

As shown in Table 1, 58 (64.4%) patients were diagnosed with HTG-AP, 20 with BAP, and 12 with AP from other causes. The mean age for all APIP patients was 29.2 ± 4.0 years old, and the mean BMI was 24.9 ± 3.28 kg/m^2^. In total, 75 (83.3%) patients were in the third trimester, 14 (15.6%) patients were in the second trimester, and 1 (1.1%) patient was in the first trimester. Of the 92 APIP patients, 64.4% were uniparous, and 35.6% were multiparous. In total, 44 (48.9%) patients had AP onset during the first pregnancy. In total, 43 (47.8%) patients were categorized as SAP, 25 (27.8%) patients with MSAP, and 22 (24.4%) patients with MAP. Moreover, 6.7% of the cases resulted in maternal death and 31.1% resulted in fetal death. In summary, the majority of APIP patients were categorized as SAP, diagnosed with HTG-AP, and had AP onset in the third trimester. In addition, all fetal deaths were intrauterine deaths.

**Table 1:** Characteristics between the fetal death and fetal survival group.

	**Total**	**Fetal death**	**Fetal survival**	***p-*value**
Number	90	28 (31.1%)	62 (68.9%)	
Age	29.2 ± 4.0	29.9 ± 4.5	29.0 ± 3.8	0.308^#^
BMI (kg/m^2^)	24.9 ± 3.28	24.9 ± 2.7	24.9 ± 3.5	0.957^#^
Gestation weeks	35.0 (26.4, 37.3)	30.1 (26.4, 35.0)	36.1 (31.7, 37.6)	0.002^a^
The first trimester	1 (1.1%)	1 (3.6%)	0 (0%)	0.074^*^
The second trimester	14 (15.6%)	7 (25.0%)	7 (11.3%)	
The third trimester	75 (83.3%)	20 (71.4%)	55 (88.7%)	
Gravidity				0.679^*^
1st pregnancy	44 (49.0%)	13 (46.4%)	31 (50.0%)	
2nd pregnancy	25 (27.8%)	7 (25.0%)	18 (29.0%)	
3rd pregnancy	14 (15.6%)	6 (21.4%)	8 (12.9%)	
4th pregnancy	4 (4.4%)	1 (3.6%)	3 (4.8%)	
Multiple pregnancy (≥ 5 times)	3 (3.3%)	1 (3.6%)	2 (3.2%)	
Parity				0.151^*^
unipara	58 (64.4%)	21 (75.0%)	38 (61.3%)	
2nd para	29 (32.2%)	7 (25.0%)	22 (35.5%)	
3rd para	2 (2.2%)	0 (0%)	2 (3.2%)	
History of abortion	22 (24.4%)	10 (35.7%)	12 (19.4%)	0.088^Δ^
Medical history				
Hypertension	3 (3.3%)	1(3.6%)	2 (3.2%)	0.678^*^
Hyperlipidemia	10 (11.1%)	4 (14.3%)	6 (9.7%)	0.377^*^
Hyperglycemia	6 (6.7%)	0 (0%)	6 (9.7%)	0.099^*^
AP/APIP history	6 (6.7%)	2 (7.1%)	4 (6.5%)	0.610^*^
Unhealthy lifestyle	2 (2.2%)	1(3.6%)	1 (1.6%)	0.528^*^
Etiology				0.794^*^
HTG	58 (64.4%)	19 (67.9%)	39 (62.9%)	
Biliary	20 (22.2%)	5 (17.9%)	15 (24.2%)	
Others	12 (13.3%)	4 (14.3%)	8 (12.9%)	
Complications before delivery				
ARDS	12 (13.3%)	8 (28.6%)	4 (6.5%)	0.007^*^
AKI	10 (11.1%)	6 (21.4%)	4 (6.5%)	0.046^*^
Shock	3 (3.3%)	2 (7.1%)	1 (1.6%)	0.678^*^
Acidosis	3 (3.3%)	3 (10.7%)	0 (0%)	0.028^*^
Severity				0.12^Δ^
MAP	22 (24.4%)	4 (14.3%)	18 (29.0%)	0.186^*^
MSAP	25 (27.8%)	6 (21.4%)	19 (30.7%)	0.366^*^
SAP	43 (47.8%)	18 (64.3%)	25 (40.3%)	0.035^*^
Systematic complications in the course		
ARDS	30 (33.3%)	15 (53.6%)	15 (24.2%)	0.006^Δ^
AKI	16 (17.8%)	9 (32.1%)	7 (11.3%)	0.017^*^
Ketoacidosis/metabolic acidosis	4 (4.4%)	3 (10.7%)	1 (1.6%)	0.088^*^
Shock	12 (13.3%)	7 (25.0%)	5 (8.1%)	0.029^*^
Sepsis	10 (11.1%)	6 (21.4%)	4 (6.5%)	0.064^*^
Ileus	7 (7.8%)	3 (10.7%)	4 (6.5%)	0.67^*^
Intestinal fistula	7 (7.8%)	4 (14.3%)	3 (4.8%)	0.198^*^
Pleural/abdominal/pelvic effusion	18 (20.0%)	6 (21.4%)	12 (19.4%)	0.82^Δ^
Thrombogenesis	6 (6.7%)	3 (10.7%)	3 (4.8%)	0.37^*^
Hemorrhage	4 (4.4%)	3 (10.7%)	1 (1.6%)	0.088^*^

*BMI, body mass index; AP, acute pancreatitis; APIP, acute pancreatitis in pregnancy; HTG, hypertriglyceridemia; ARDS, acute respiratory distress syndrome; AKI, acute kidney injury; p-values marked by*

Δ *were analyzed by Pearson chi-square tests and*

* *were analyzed by Fisher's exact tests. p-Values were analyzed by Student t-tests marked by*

# *and Mann–Whitney U-tests marked by ^a^*.

### The Fetal Death Group Had Fewer Gestation Weeks, More Pre-delivery Systematic Complications, and Higher Rates of Severe Acute Pancreatitis

Of the 90 APIP patients, 28 (31.1%) had fetal death and 62 (68.9%) had fetal survival. As shown in Table 1, the clinical features were analyzed between the fetal death and survival groups in the pre-delivery period. The results show that the fetal death group had a significant lower gestation week (30.1 *vs*. 36.1 weeks, *p* = 0.002), more pre-delivery systematic complications such as ARDS (28.6 *vs*. 6.5%, *p* = 0.007), AKI (21.4 *vs*. 6.5%, *p* = 0.046), and acidosis (10.7 *vs*. 0, *p* = 0.028), and a higher rate of SAP than the fetal survival group (64.3 *vs*. 40.3%, *p* = 0.035). Moreover, the fetal death group tended to have more systematic complications, such as ARDS (*p* = 0.006), AKI (*p* = 0.017), and shock (*p* = 0.029), during the entire AP course.

### Gestation Week and Pre-delivery Acute Respiratory Distress Syndrome Were Independent Risk Factors for Fetal Intrauterine Death

As shown in Table 2, the multivariable logistic regression analysis found that the pre-delivery systematic complication of ARDS (OR, 5.8; 95% CI, 1.5–22.4; *p* = 0.010) and gestational week (OR, 0.9; 95% CI, 0.8–1.0; *p* = 0.010) were independent risk factors for fetal death. A goodness of fit of the model was performed, and the result of Hosmer and Lemeshow test was 9.206 (df = 8. *p* = 0.325).

**Table 2:** Multivariable logistic regression analysis of risk factors of fetal intrauterine death in the period of pre-delivery.

**Risk factors**	**OR (95% CI)**	***p-*value**
Gestation weeks	0.9 (0.8–1.0)	0.011
Pre-delivery ARDS	5.8 (1.5–22.4)	0.010

*OR, odds ratio; ARDS, acute respiratory distress syndrome*.

### Gestation Week and Fetal Intrauterine Death Were Risk Factors for Acute Pancreatitis Severity

Based on the severity of maternal AP, the 90 APIP patients were divided into two groups: the SAP group and the non-SAP group (MAP/MSAP). As shown in Table 3 compared with the non-SAP group, the SAP group had higher gestation age (35.4 vs. 32.4 weeks, p = 0.010) and more fetal death (41.9 *vs*. 21.3%, *p* = 0.035). As shown in Table 4, gestational week (OR, 1.2; 95% CI, 1.1–1.3; *p* = 0.003) and fetal intrauterine death (OR, 5.9; 95% CI, 1.8–19.4; *p* = 0.003) were found to be independent risk factors for maternal AP severity. A goodness of fit of the model was performed, and the result of Hosmer and Lemeshow test was 12.813 (df = 8. *p* = 0.118).

**Table 3:** Comparison between MAP/MSAP and SAP patients.

	**MAP/MSAP**	**SAP**	***p*-value**
Number	47 (52.2%)	43 (47.8%)	
Age	29.3 ± 4.2	29.2 ± 3.9	0.937^#^
BMI	24.9 ± 2.6	24.8 ± 3.8	0.933^#^
Gestation weeks	32.4 (27.6, 37.3)	35.4 (32.3, 37.3)	0.010^a^
The first trimester	1 (2.1%)	0 (0%)	0.52^*^
The second trimester	11 (23.4%)	3 (7.0%)	0.032^*^
The third trimester	35 (74.5%)	40 (93.0%)	0.018^Δ^
Gravidity			0.816^*^
1st pregnancy	24 (51.1%)	20 (46.5%)	
2nd pregnancy	12 (25.5%)	13 (30.2%)	
3rd pregnancy	7 (14.9%)	7 (16.3%)	
4th pregnancy	3 (6.4%)	1 (2.3%)	
Multiple pregnancy(≥ 5 times)	1 (2.1%)	2 (4.7%)	
Parity			0.952^*^
Unipara	31 (66.0%)	27 (62.8%)	
2nd para	15 (31.9%)	15 (3.5%)	
3rd para	1 (2.1%)	1 (2.3%)	
History of abortion	11 (23.4%)	11 (25.6%)	0.81^Δ^
Medical history			
Hypertension	2 (4.3%)	1 (2.3%)	0.53^*^
Hyperlipidemia	6 (12.8%)	4 (9.3%)	0.74^*^
Hyperglycemia	5 (10.6%)	1 (2.3%)	0.21^*^
AP/APIP history	3 (6.4%)	3 (7.0%)	0.62^*^
Unhealthy lifestyle	2 (4.3%)	0 (0%)	0.27^*^
Etiology			0.421^Δ^
HTG	33 (70.2%)	25 (58.1%)	
Biliary	8 (17.0%)	12 (27.9%)	
Others	6 (12.8%)	6 (14.0%)	
Fetal intrauterine death	10 (21.3%)	18 (41.9%)	0.035^Δ^

*MAP, mild acute pancreatitis; MSAP, moderate-severe acute pancreatitis; SAP, severe acute pancreatitis; BMI, body mass index; AP, acute pancreatitis; APIP, acute pancreatitis in pregnancy; HTG, hypertriglyceridemia; p-values marked by*

Δ *were analyzed by Pearson chi-square tests and*

* *were analyzed by Fisher's exact tests. p-Values were analyzed by Student t-tests marked by*

# *and Mann–Whitney U-tests marked by ^a^*.

**Table 4:** Multivariable logistic regression for SAP.

	**OR (95% CI)**	***p*-value**
Gestation weeks	1.2 (1.1–1.3)	0.003
Fetal intrauterine death	5.9 (1.8–19.4)	0.003

*SAP, severe acute pancreatitis; OR, odds ratio*.

## Discussion

APIP is a rare but critical disease with a high fetal fatality rate which is even higher in China than in other countries. Various studies have shown that the fetal mortality in cases of APIP is <10% abroad, with 4.7% fetal mortality in 43 APIP patients (6), 3.6% fetal mortality in 101 APIP patients (5), and 6.6% fetal mortality in 103 APIP patients (4). However, the fetal mortality rate is approximately 2.56–31.58% in China, as Li et al. reported 8% fetal mortality in 25 APIP patients (9), Huang et al. reported 9.5% in 21 patients (23), Xu et al. reported 19.4% in 36 patients (13), Zhang et al. reported 31.58% in 38 patients (2), and Luo et al. reported 11.6% in 121 patients (24). In the previous study, our center reported a fetal mortality rate of 23.19% in 69 patients with APIP (17).

Herein, we identified 90 APIP patients from 2012 to 2020 and reported that fetal mortality was 31.1% (28/90), with five fetal deaths after being transferred to our center and 23 fetal deaths before transfer. First, our results show that gestational age was an independent risk factor for both fetal intrauterine death and the severity of maternal AP. As reported in our previous research, the mean gestation week for the fetal death group was 32.7 ± 4.7 weeks, while the fetal survival group was 36.4 ± 2.2 weeks (25). Other studies also reported that earlier gestation week was related to higher fetal mortality (4, 17, 26). For example, Tang et al. reported that six patients suffered fetal deaths in the first or second trimester (4), and Xu et al. reported that five patients had fetal deaths in the first or second trimester (13). Moreover, the majority of fetal deaths in cases of APIP were fetal intrauterine deaths. Our previous study reported that 75% of the patients had fetal intrauterine deaths (17). Eddy et al. reported fetal intrauterine death in all fetal deaths (5) and Zhang et al. reported 83.3% fetal intrauterine deaths in a total of 12 fetal deaths (2).

In addition, for the first time, the risk factors for fetal intrauterine death were analyzed among the clinical features in the pre-delivery period. The results show that pre-delivery ARDS was an independent risk factor for fetal intrauterine death, together with gestational age. ARDS is a complex disease characterized by hypoxemia caused by inflammation-induced injury to the alveolar–capillary barrier. Adult ARDS in pregnancy mostly occurs due to infection, preeclampsia, eclampsia, and aspiration (27, 28). Very few studies on adult ARDS during pregnancy are also concerned with AP. Maternal ARDS patients have been reported to have a high rate of fetal death, spontaneous preterm labor, and fetal heart rate abnormalities. Animal model research has found that cases of APIP in rats had pro-inflammatory cytokines and oxidative stress involved in the development of systemic complications such as lung and renal damage (29–32). ARDS is a critical concern in fetal intrauterine death because of the lack of uteroplacental perfusion, pro-inflammatory cytokines, and oxidative stress. When ARDS occurs, along with central hypovolemia, uteroplacental perfusion is usually compromised and may be catastrophic to the health of the fetus (33). In these cases, the matrix is in a state of chronic respiratory alkalosis, resulting in a decreased buffering capacity for any additional metabolic acidosis during the gestational period (34). All of these symptoms have adverse effects confirmed by the positive effect of timely termination of the pregnancy. Some retrospective analysis has suggested that delivery is an alternative for women with ARDS, which might be helpful for improving maternal health status (35).

Finally, we analyzed the interaction between mother and fetus, and found that fetal intrauterine death was also an independent risk factor for maternal AP severity. There were significantly more SAP patients in the fetal death group (*p* = 0.035). Fetal intrauterine death results in a worse AP course and more systematic complications. Previous studies have suggested that fetal death is associated with an increase in severe maternal diseases such as acute myocardial infarction, amniotic fluid embolism, and preeclampsia or eclampsia (36–38). However, there have been no studies on the influence of fetal death on maternal outcomes in cases of APIP. In this study, we first analyzed the interaction of fetal intrauterine death with the severity of maternal AP and found that fetal intrauterine death was an independent risk factor for maternal AP severity, together with gestational week.

There were some limitations to this study. First, the localized complications were evaluated by contrast-enhanced CT or MRI, and all were performed after delivery. Thus, it was difficult to evaluate the AP severity before delivery and to distinguish whether the severity progressed after fetal intrauterine loss. Second, as one of the largest acute pancreatitis transfer centers in China, the majority of patients were diagnosed with SAP (47.78%) and HTG-APIP (64.4%). Tang et al. and Yang et al. report that HTG is a risk factor for fetal death in APIP (8, 26). Compared with other etiologies, HTG-APIP was more prevalent in severe AP cases, with more complications, and poorer prognosis (7, 13, 16, 17). The majority of our patients were HTG-APIP with severe AP, resulting in fetal loss. This might be the reason why the etiology was not a risk factor for fetal intrauterine death.

## Conclusion

Pre-delivery ARDS and gestational age were found to be independent risk factors for fetal intrauterine death. Fetal intrauterine death and gestational week were independent risk factors for maternal AP severity.

## Data Availability Statement

The raw data supporting the conclusions of this article will be made available by the authors, without undue reservation.

## Ethics Statement

The studies involving human participants were reviewed and approved by Acute Pancreatitis Database Management Committee. Written informed consent for participation was not required for this study in accordance with the national legislation and the institutional requirements.

## Author Contributions

ZT, QY, XS, and NP conceived and designed the study. XS, YH, NP, and GZ collected the data and performed the analysis. JZha, JZho, BY, GL, ZT, and WL treated the admitted patients. XS, NP, QY, and ZT drafted the paper. YL, LK, and WL critically revised the manuscript. All authors have read and approved the manuscript.

## Funding

This study was supported by the National Natural Science Foundation of China (Nos. 81870441, 81900592, and 82070669).

## Conflict of Interest

The authors declare that the research was conducted in the absence of any commercial or financial relationships that could be construed as a potential conflict of interest.

## Publisher's Note

All claims expressed in this article are solely those of the authors and do not necessarily represent those of their affiliated organizations, or those of the publisher, the editors and the reviewers. Any product that may be evaluated in this article, or claim that may be made by its manufacturer, is not guaranteed or endorsed by the publisher.
